# Phylogeographic dynamics of the arthropod vector, the blacklegged tick (*Ixodes scapularis*)

**DOI:** 10.1186/s13071-022-05304-9

**Published:** 2022-06-28

**Authors:** Kayleigh R. O’Keeffe, Zachary J. Oppler, Melissa Prusinski, Richard C. Falco, JoAnne Oliver, Jamie Haight, Lee Ann Sporn, P. Bryon Backenson, Dustin Brisson

**Affiliations:** 1 Department of Biology, University of Pennsylvania, Philadelphia, PA USA; 2 New York State Department of Health, Albany, NY USA; 3 Department of Health, Central New York Regional Office, Syracuse, NY 13202 USA; 4 Paul Smith’s College, Paul Smiths, NY USA

**Keywords:** Ticks, Vectors, Lyme disease, Phylogeography, Migration, Population structure

## Abstract

**Background:**

The emergence of vector-borne pathogens in novel geographic areas is regulated by the migration of their arthropod vectors. Blacklegged ticks (*Ixodes scapularis*) and the pathogens they vector, including the causative agents of Lyme disease, babesiosis and anaplasmosis, continue to grow in their population sizes and to expand in geographic range. Migration of this vector over the previous decades has been implicated as the cause of the re-emergence of the most prevalent infectious diseases in North America.

**Methods:**

We systematically collected ticks from across New York State (hereafter referred to as New York) from 2004 to 2017 as part of routine tick-borne pathogen surveillance in the state. This time frame corresponds with an increase in range and incidence of tick-borne diseases within New York. We randomly sampled ticks from this collection to explore the evolutionary history and population dynamics of *I. scapularis*. We sequenced the mitochondrial genomes of each tick to characterize their current and historical spatial genetic structure and population growth using phylogeographic methods.

**Results:**

We sequenced whole mitochondrial genomes from 277 ticks collected across New York between 2004 and 2017. We found evidence of population genetic structure at a broad geographic scale due to differences in the relative abundance, but not the composition, of haplotypes among sampled ticks. Ticks were often most closely related to ticks from the same and nearby collection sites. The data indicate that both short- and long-range migration events shape the population dynamics of blacklegged ticks in New York.

**Conclusions:**

We detailed the population dynamics of the blacklegged tick (*Ixodes scapularis*) in New York during a time frame in which tick-borne diseases were increasing in range and incidence. Migration of ticks occurred at both coarse and fine scales in the recent past despite evidence of limits to gene flow. Past and current tick population dynamics have implications for further range expansion as habitat suitability for ticks changes due to global climate change. Analyses of mitochondrial genome sequencing data will expound upon previously identified drivers of tick presence and abundance as well as identify additional drivers. These data provide a foundation on which to generate testable hypotheses on the drivers of tick population dynamics occurring at finer scales.

**Graphical Abstract:**

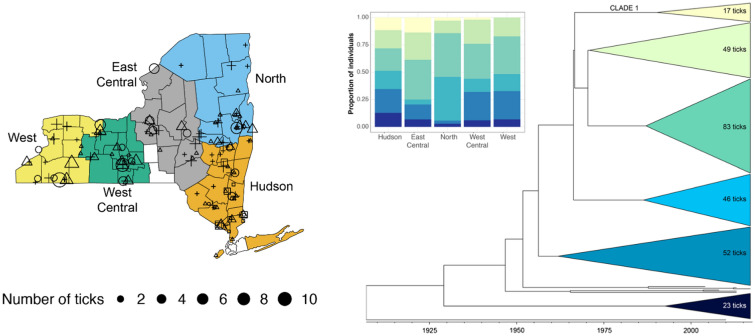

**Supplementary Information:**

The online version contains supplementary material available at 10.1186/s13071-022-05304-9.

## Background

Vector-borne diseases are the most common type of emerging and re-emerging infectious diseases (EIDs) in the world and constitute major threats to public health [1, 2]. The recent surge in vector-borne diseases is a consequence of the effects of global climate change and human land-use change on arthropod vector population dynamics [3–9]. The distribution and abundance of vectors are major drivers in vector-borne disease spread [10, 11]. Therefore, identifying the ecological processes governing arthropod vector population dynamics is essential for predicting future spread of diseases caused by vector-borne pathogens [12]. Such investigations are challenging as they require a wealth of fine-scale temporal and spatial data that encompass a time frame in which demographic processes occurred [13–15]. Here, we address this challenge using samples of blacklegged ticks (*Ixodes scapularis*) collected across New York State (hereafter referred to as New York) during a time frame of demographic dynamics to delineate the patterns of tick population dynamics over time and space.

*Ixodes scapularis* is the most important vector of EIDs in the United States [16], with this tick vectoring pathogens that cause numerous diseases, such as Lyme disease, human babesiosis and anaplasmosis [17]. The population density and geographic range of *I. scapularis* have increased in the northeastern United States over recent decades [10, 18–21]. For example, the range of ticks in New York has expanded markedly into the western and northern parts of the state during the last decade alone [22].

Tick population dynamics are a primary driver of the population dynamics of tick-transmitted pathogens. For example, changes in the population demography of ticks in the Hudson River Valley explained over 40% of changes in human Lyme disease incidence in the same area and time frame [12]. Investigating tick population dynamics across broad geographic and temporal ranges with sufficient analytical power to identify the ecological processes underlying changes in tick populations is critical to the design of effective control strategies [23–30]. However, the majority of studies have been limited in geographic and temporal range due to the difficulty of obtaining samples at the appropriate temporal and spatial scales [10, 12]. Exploratory analyses that delineate tick population dynamics at coarse scales provide the foundation to generate hypotheses about the fine-scale ecological processes that influence tick and tick-borne pathogen populations. Subsequent analyses of targeted samples collected at finer scales are necessary to address these hypotheses.

Exploratory research has been the cornerstone for hypothesis generation across scientific fields and is key to furthering our understanding of vector biology. For example, genome-wide association studies (GWAS) have facilitated the generation of numerous hypotheses about the function of multiple genes in traits and diseases, which have then been tested in subsequent functional investigations [31–33]. Exploratory research, such as GWAS, that facilitates broad-scale inferences and hypothesis generation are feasible due to the increase in the availability of massive datasets on genomes, species distributions, climate, land cover and other types of ‘big data’ [34, 35]. By taking a similar exploratory approach with an extensive sample collection of ticks, we characterized natural vector population dynamics at broad spatial scales to generate hypotheses on how biological, environmental and human features impact such dynamics.

In this study, we describe the population dynamic patterns of blacklegged ticks in New York using a sample collection that represents a time frame and geographic range in which tick populations and tick-borne disease incidence has grown and expanded. Specifically, we analyzed the mitochondrial genomes from 277 *I. scapularis* ticks collected across the state of New York from 2004–2017. We inferred tick population dynamics and generated hypotheses about the mechanisms underlying those dynamics using the additional statistical power derived from a sample selection representing state-wide environmental variation and abundant genetic information from each tick. Specifically, we (1) characterized population genetic structure and (2) characterized population growth. This work is critical to designing effective control and management strategies for ticks and tick-borne diseases.

## Methods

### Population sampling

Host-seeking ticks have been collected since 2003 using standardized flagging methods [36] as part of routine tick-borne pathogen surveillance in New York [37]. This collection includes *I. scapularis* ticks and their associated pathogens from 667 sites [22, 37]. Some locations were sampled annually while others were sampled on a rotational basis every 2 to 5 years. Collections have been systematic with respect to both space and time. Collected ticks were stored in 80–100% ethanol at 4 ºC until sorted by developmental stage and confirmed to be *I. scapularis* using dichotomous keys [38, 39]. Following initial processing, individual ticks were placed in 1.5-ml Eppendorf tubes and preserved in 80–100% ethanol at − 20 ºC until DNA extraction.

**Fig. 1:**
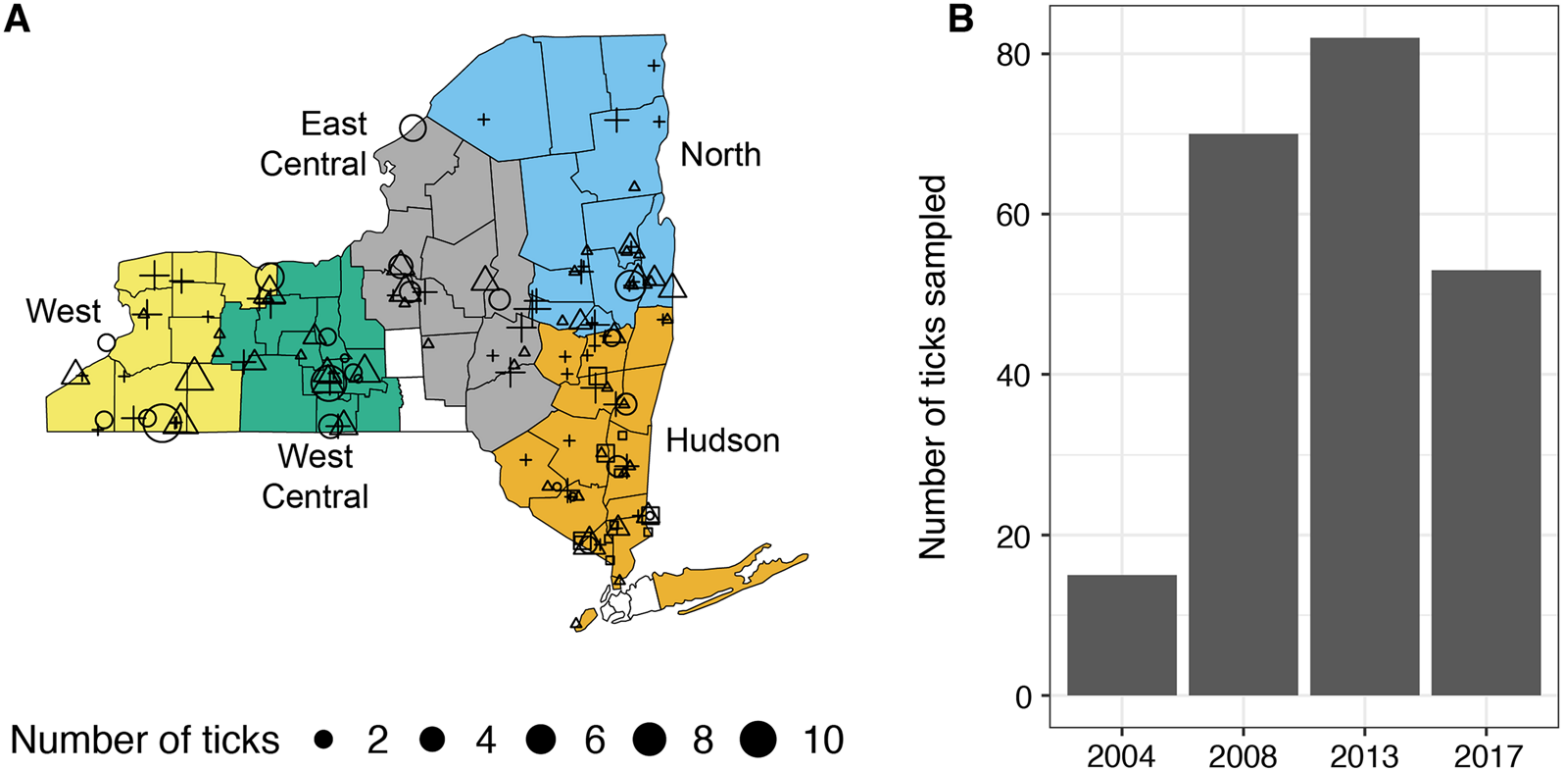
*Ixodes scapularis* ticks were collected across New York State (‘New York’) during a time period in which tick populations were growing and expanding. **A** Geographic distribution of the 277 ticks collected in New York. The year in which samples were collected is denoted by the shape of the symbol, with squares, circles, triangles and crosses indicating ticks collected in 2004, 2008, 2013 and 2017, respectively. The size of the symbol corresponds to the number of ticks analyzed from a given site. The ticks analyzed represent a stratified random sample from five color-coded regions within New York. *I. scapularis* ticks were not collected during all study years from the following counties: Lewis, Franklin, Broome, Nassau, Suffolk and Cortland. **B** Distribution of samples collected per year

We randomly sampled 400 ticks from this collection to investigate current and historical patterns of population growth and gene flow across New York. We employed a stratified random sample design to maximize spatial coverage while minimizing violations of the assumptions of the subsequent analyses. We divided New York into five approximately equally-sized regions (Fig. 1a; these regions are referred to in this article as Hudson/Hudson River Valley, North, East Central, West Central and West). From each region, we randomly sampled ticks collected in each of three different years (2008, 2013 and 2017). Specifically, 25 ticks were sampled from each region and year combination. In addition, 25 ticks were sampled from those collected in 2004 from the Hudson River Valley, the only region in which ticks were collected prior to the initiation of state-wide surveillance in 2008. At least one tick was randomly sampled from each county when available to maximize the spatial distribution of the samples within each region. Sample metadata, including sampling year, location, region, developmental stage, and sex, are listed in Additional file 1: Table S1.

Several ticks from outside of New York were included to serve as geographic outgroups. Specifically, we included three ticks collected from southeastern Pennsylvania (2 collected in 2009, 1 in 2010 from Crow's Nest Preserve [Natural Lands Trust], a deciduous mid-Atlantic forest with interspersed agricultural lands [40]) in Chester County (40°11′ N, 75°17′ W), and three ticks collected from Loudoun County, Virginia in 2017 (collected by Jory Brinkerhoff at the University of Richmond).

### DNA extractions

Total genomic DNA was extracted from individual *I. scapularis* ticks as previously described [37]. Briefly, ticks were double rinsed with nuclease-free distilled water and placed in 2.0-ml round-bottomed Safe-Lock tubes containing two 4-mm stainless steel grinding beads and 180 µl phosphate buffered saline (PBS), pH 7.2. Samples were ground using an electric tissue homogenizer. Whole genomic DNA was extracted from homogenized samples using the DNeasy™ Blood and Tissue kit (Qiagen, Hilden, Germany) following the manufacturer’s recommendations (Purification of Total DNA from Insects protocol). DNA from individual ticks was initially eluted in 200 µl ultra-pure water and stored at – 20 ºC. In the case of the 2017 samples, second elutions of 100 µl ultra-pure water were used for sequencing.

### Mitochondrial genome amplification and sequencing

We designed primers for long-range PCR to capture the entire mitochondrial genome of each tick. Briefly, we developed primers that amplify the mitochondrial genome in four separate but overlapping fragments (Additional file 2). We used the *I. ricinus* reference genome as the basis for primer design with the NCBI primer design tool [41]. We chose primer pairs that fell within regions identified as highly conserved and slightly overlapped one another. Primer sequences are reported in Additional file 2: Table S2. Each fragment was amplified separately using the same PCR program. Each 50-µL PCR reaction volume contained 1 µl GXL polymerase (TaKaRa Bio, Kusatsu, Shiga, Japan), 10 µl of 5× GXL PCR buffer, 4 µl GXL dNTPs, 5 µl forward primer, 5 µl reverse primer, 15 µl water and 10 µl DNA. The cycling conditions were: initial denaturation at 95 °C for 30 s; followed by 5 cycles of 98 ºC for 10 s, 64 ºC for 15 s, 68 ºC for 4.5 min, 5 cycles of 98ºC for 10 s, 67 ºC for 15 s, 68 ºC for 4.5 min and 30 cycles of 98ºC for 10 s, 70 ºC for 15 s, 68 ºC for 4.5 min. PCR product quality was determined by visualizing products using gel electrophoresis and quantifying concentration using Qubit. PCR product concentrations were normalized to 10 ng/µl and pooled in one run on an Illumina MiSeq instrument (Illumina, San Diego, CA, USA) using a paired-end 2 × 300 bp kit (Illumina).

### Read alignment and SNP detection

Reads were demultiplexed following Illumina’s Generate FASTQ workflow and trimmed with CutAdapt (version 1.18 [42]). We aligned reads to both the *I. ricinus* [43] and *Ixodes scapularis* [44] mitochondrial genomes using BWA mem (v. 0.7.17 [45, 46]). The resulting set of variant calling files were 99.2% identical, and there was no difference in between results of subsequent analyses. Duplicate reads were excluded from downstream analysis using the Picard Suite (v. 2.18.16 [47]) MarkDuplicates. Only samples with mean coverage > 10× were retained for analysis. Single nucleotides that differed from the draft genome were identified with GATK (v. 4.2.0 [48, 49]) HaplotypeCaller (ploidy set to 1) to generate sample-specific gVCF files. We conducted joint genotyping of samples with GATK GenotypeGVCFs. We excluded indels and single nucleotide polymorphisms (SNPs) with signals of low mapping or genotyping quality with GATK VariantFiltration using filters recommended by GATK: quality by depth (QD < 2.0), Fisher strand bias (FS > 60.0), mapping quality (MQ < 40.0), mapping quality rank sum test (MQRankSum <  − 12.5), the Mann–Whitney rank sum test (ReadPosRankSum <  − 8.0), low genotype call (GQ < 20) and strand odds ratio test (SOR > 4.0). Filtered genotypes were set to no call (GATK option: setFilteredGtToNocall). We used the GATK tool FastaAlternateReferenceMaker to create an alignment of consensus sequences from the variant file. We accounted for no calls within each sample in two ways: (i) in one set of consensus files, reference bases were used where there was no call made; (ii) in another set of consensus files, no calls were marked with *N*s. FastaAlternateReferenceMaker outputs consensus files use reference bases where no calls are made by default. To mark no calls with *N*s, we used SelectVariants to select sites in which no call could be made, and the resulting VCF file was used as a mask file (using the snp-mask parameter) when running FastaAlternateReferenceMaker. This resulted in two sets of alignments of consensus sequences of the mitochondria (14,420 bp). We ran all analyses with both sets of files, and found that our inferences were robust to the method used. We report the results with *N*s in the main text.

### Phylogeographic analyses

We used a coalescent approach implemented in BEAST 2 (v. 2.6.3 [50]) to infer the phylogeographic history of *I. scapularis*. BEAST estimates the phylogenetic topology while simultaneously inferring the geographic location of ancestral nodes, migratory events among locations and demography. Recombination-free alignments were used to fit two alternative models, including either a strict molecular clock or a relaxed (uncorrelated log-normal) molecular clock (allowing substitution rates to vary across tree branches). Each model included a Bayesian skyline model of changing population size, an Hasegawa-Kishino-Yano (HKY) substitution model and gamma-distributed rate variation across sites.

We ran chains of 500 million iterations or until convergence for each model. Chains were thinned by sampling every 50,000 iterations, and 10% of each chain was discarded as burn-in. Tracer v. 1.7.1 was used to assess convergence and confirm effective sample sizes were > 200 for each parameter [51]. We used path sampling to estimate the marginal likelihood of each model to identify the best-fitting model [52]. We compared alternative models with Bayes factors and identified the relaxed log-normal clock as the best-fitting model used in all above analyses. Maximum clade credibility (MCC) trees were generated using TreeAnnotator [53], and trees were visualized in FigTree (http://tree.bio.ed.ac.uk/software/figtree/).

We complemented the coalescent-based approach with a mismatch distribution analysis implemented in DnaSP v5 [54]. We tested if our observed data were consistent with a mismatch distribution expected from a sudden population expansion or a spatial expansion. The observed mismatch distribution and expected mismatch distribution for a stable population were visualized in the ggplot2 package (v. 3.3.3 [55]) in R v. 3.6.1.

### Population demography

We quantified pairwise genetic distances among samples using the Kimura 2-parameter model [56] in MEGA X [57] to investigate genetic differentiation. Genetic differentiation was estimated both within regions and between regions by comparing pairwise genetic distances between ticks. We used linear models with R (version 3.6.1) to compare within-region pairwise distances across regions and over time (Distance ~ Region × Year) and between-region pairwise distances across region pairs (Distance ~ Region Pair). We used the package *emmeans* (version 1.3.2 [58]) to evaluate the estimated marginal means of diversity indices for each explanatory variable level, adjusted for multiple comparisons (Tukey's honest significant difference [HSD]).

To test for isolation by distance [59], we performed a Mantel permutation test [60] with 9999 permutations of geographic distance between collection sites and genetic distance as quantified by the Kimura 2-parameter model. The haversine distance between ticks was calculated from the longitude and latitude of tick collection sites using the *distm* function within the vegan library in R [61]. We performed the Mantel test using the *mantel* function within vegan. The Mantel statistic, r_M_, quantifies the correlation between genetic and geographic distance.

## Results

### Field sampling and variation

We sequenced whole mitochondrial genomes from 277 ticks collected in New York (Table 1; Fig. 1). The 187 identified SNPs resulted in 88 unique haplotypes among New York ticks (Table 1). The number of haplotypes and haplotype diversity were highest in the Hudson River Valley (Hudson region) and lowest in the Northern region of New York after correcting for differences in sample sizes. The six ticks from outside of New York (3 from Virginia, 3 from Pennsylvania) had nine additional SNPs, resulting in an additional two haplotypes.

**Table 1:** Genetic diversity indices including the number of haplotypes and haplotype diversity of *Ixodes scapularis* for each of the five regions sampled in New York State

Genetic diversity indices	Regions				
	Hudson	North	East Central	West Central	West
Sample size (*n* ticks)	79	35	44	51	62
H	48	22	28	33	40
H_d_	0.983	0.965	0.970	0.966	0.981

*H* Number of haplotypes,* H*_*d*_ haplotype diversity

### Population genetic structure among ticks in New York

**Fig. 2:**
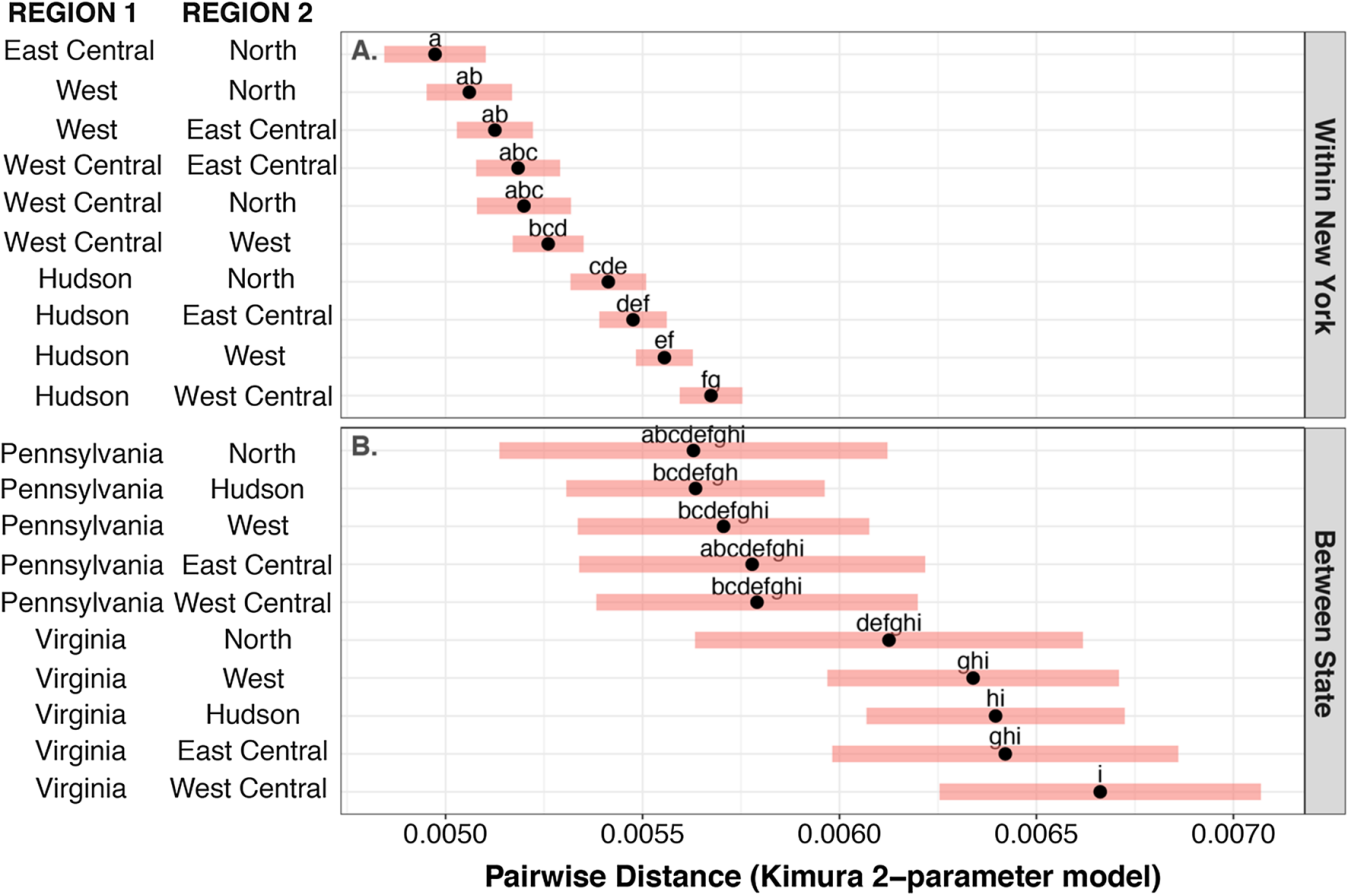
Population genetic differences among regions increase with increasing geographic distance. Points are estimated marginal means with red bands indicating ± 95% confidence intervals. Lowercase letters mark statistical differences in pairwise distances (using Kimura’s 2-parameter model) evaluated with Tukey’s honest significant difference (HSD). **A** Pairwise distances between ticks from the different regions within New York are generally low, consistent with weak population genetic structure. Yet, comparisons between the Hudson River Valley (Hudson) and any other regions within New York are greater than all other between-region comparisons. **B** Pairwise distances between ticks from outside of New York and ticks from any region within New York are greater than average pairwise distances between ticks from different regions within New York

There was clear evidence of population genetic structure among the five regions delineated within New York. That is, all F_st_ values (measure of pairwise population differentiation due to genetic structure) were statistically significant except one, indicating restricted gene flow among the sampled regions (Table 2). The average pairwise genetic distances between ticks from the Hudson River Valley and ticks from any other of the sampled regions within New York were distinctly larger than all other between-region comparisons (Fig. 2a). Notably, ticks from the Hudson River Valley region were most distinct from ticks collected in the regions that were most distant to the Hudson River Valley (West and West Central regions).

**Table 2:** Population genetic structure of *Ixodes scapularis* among sampled regions of New York State

Regions^a^	Regions^a^				
	Hudson (48)	North (22)	East Central (28)	West Central (33)	West (40)
Hudson (48)	–	0.016	0.011	0.009	0.007
North (22)	11/59	–	0.014	0.017	0.016
East Central (28)	13/63	5/45	–	0.009	0.012
West Central (33)	16/65	9/46	13/48	–	*0.006*
West (40)	12/76	7/55	11/57	17/56	–

^a^Pairwise F_st_ values ((measure of pairwise population differentiation due to genetic structure)) are presented above the diagonal (single italicized value [region West Central] is not statistically significant). The total number of haplotypes from each region is given in parentheses in the row and column headers. The number of haplotypes shared among pairs of locations over the total number of haplotypes from a given pair of regions is presented below the diagonal

**Fig. 3:**
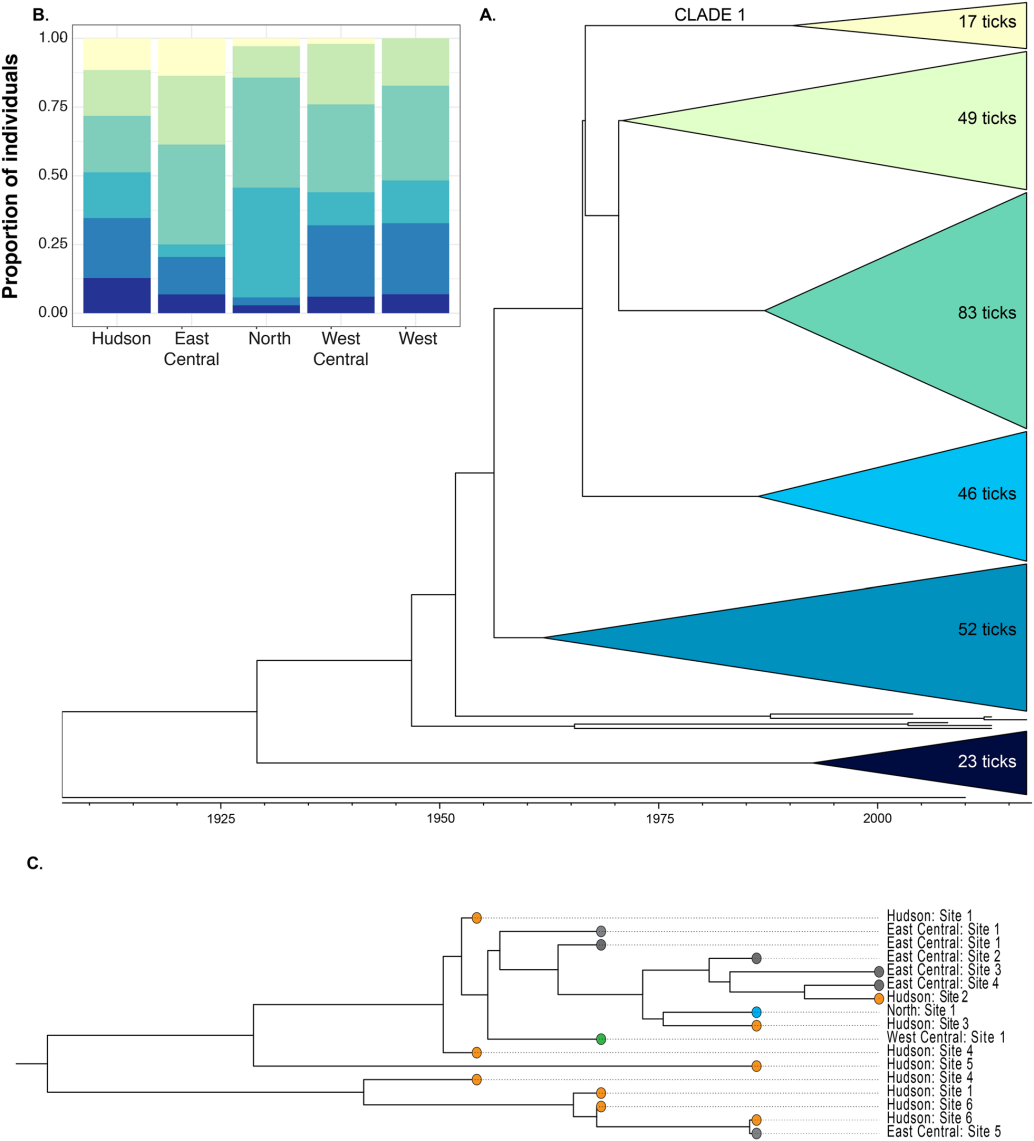
Relative abundance, not composition, of haplotypes of *I. scapularis* varies among regions. **A** Although closely related ticks could be found across New York, ticks within each clade tended to cluster geographically, suggesting isolation by distance at fine spatial scales. Dated maximum clade credibility (MCC) phylogeny of the best-fitting model inferred with BEAST (strict molecular clock model; Bayesian extended skyline population size) of *I. scapularis* in NY. Tips are collapsed into major clades in order to depict coarse scale patterns. Each clade is labeled with the number of ticks it contains. **B** Bar chart depicts the proportion of ticks from each clade found in each region. At least one tick from each clade was present within each region except that the Clade 1 is not present in the North region. Importantly, the proportion of ticks from each clade varied across New York regions. Ticks collected in the Hudson River Valley were distributed relatively evenly among the phylogenetic clades, whereas ticks from only two clades dominated in the North region. **C** Although closely related ticks could be found across New York, ticks within each clade tended to cluster geographically, suggesting isolation by distance at fine spatial scales. Clade 1 of the dated phylogeny of *I. scapularis* in New York with tips are colored by sampling region. In this example, ticks from four of the five regions were represented with unmistakable geographic clustering. That is, ticks from the East Central region were most closely related to each other while ticks from the Hudson River Valley were most closely related to each other

The population genetic structure among ticks from the different regions of New York was driven by differences in the relative frequency of haplotypes rather than by differences in which haplotypes are present. Ticks from New York can be divided into six major phylogenetic clades which do not strictly reflect geography (Fig. 3a). At least one tick from each clade was collected from each geographic region except Clade 1, which was not represented in the West region (Fig. 3b). Similarly, regions shared high proportions of haplotypes (Table 2) and clusters of haplotypes (determined by K-means clustering) were represented across all regions for all values of K tested (Additional file 3: Figure S1). However, the relative frequency of ticks from each phylogenetic clade, K-means clusters and haplotypes varies among regions. For example, the Hudson River Valley had approximately equal proportions of ticks from each phylogenetic clade while the North region was dominated by ticks from two clades.

At a finer geographic scale, ticks collected from the same site were often more closely related to each other than to ticks from different sites (Fig. 3c). There was also some evidence of a correlation between geographic distance between sites where ticks were collected and genetic distance between those ticks (Mantel test,* P* = 0.061), although the relationship is weak (*r* = 0.032).

### Population genetic structure within and beyond New York

Isolation by distance was further supported in analyses that included ticks collected in the states of Pennsylvania and Virginia. The average pairwise genetic distances between ticks from different regions was positively correlated with the geographic distances between those regions. Specifically, average pairwise genetic distances were highest between ticks collected in regions of New York and those collected from Virginia; the second highest pairwise genetic distances were between ticks collected in regions of New York and those collected from Pennsylvania (Fig. 2b). When ticks from outside of New York were included in the analyses, the correlation between geographic distance and genetic distance had more statistical support (Mantel test* P* = 0.042;* r* = 0.045). While one tick from Pennsylvania was an outgroup to the phylogeny of all ticks included in this study, the other ticks from outside New York were not. These ticks represented unique haplotypes but shared SNPs with ticks sampled from New York, consistent with a recent shared history of all sampled ticks.

**Fig. 4:**
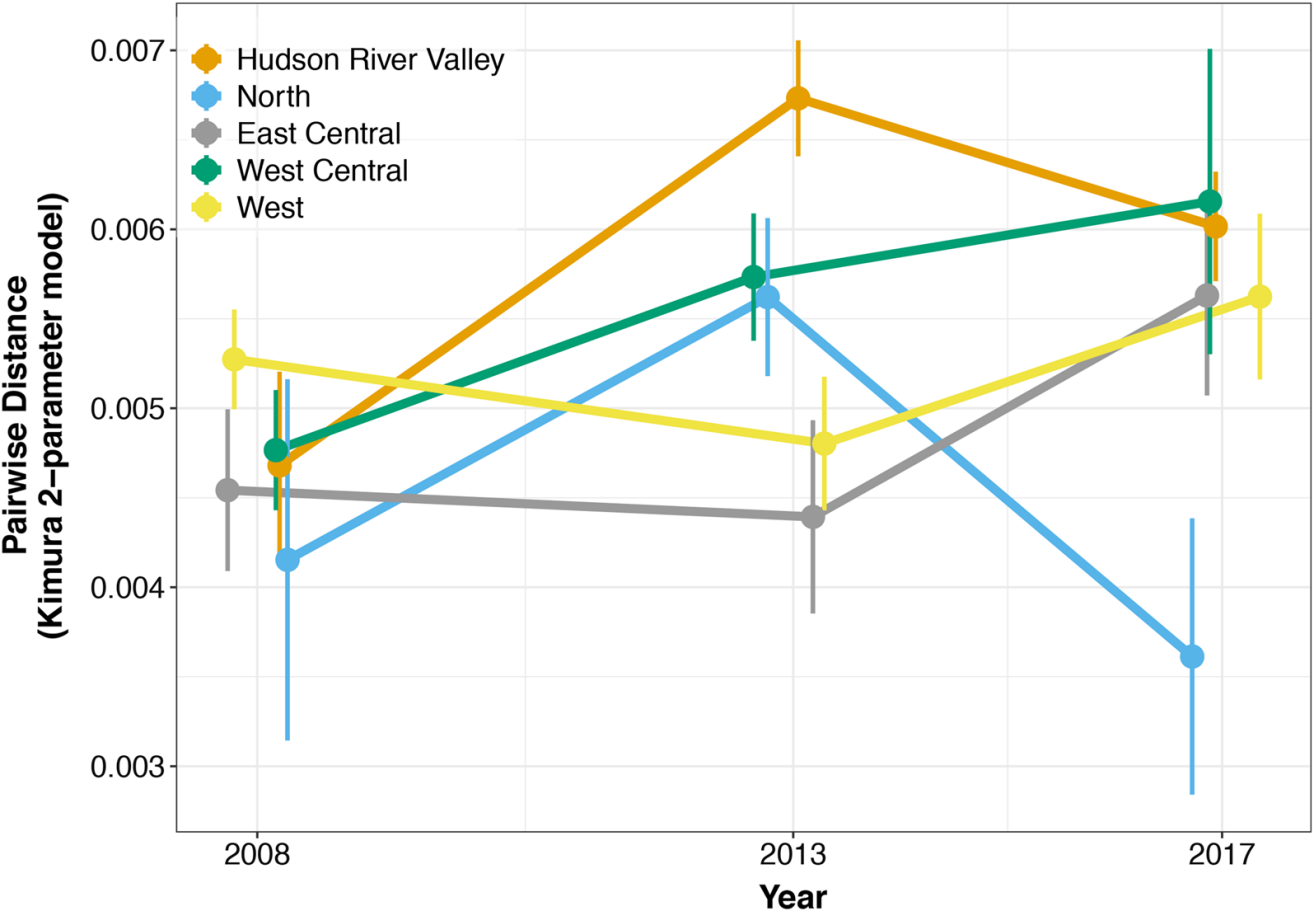
Within-region population genetic diversity varies across space and time. Points represent the average pairwise distance (using the Kimura 2-parameter model) between ticks within each of the five regions of New York in each of three collection years. Vertical lines around each point represent ± 95% confidence intervals and lines connect points from the same region across sampling years. The year 2004 was excluded because ticks were sampled only in the Hudson River Valley that year. Points are jittered on the* X*-axis for clarity. In 2008, the first year in which ticks were collected from all regions, the population genetic diversity did not differ among regions. Within-region diversity was generally high in the Hudson River Valley in subsequent years, and clearly higher than other regions among ticks collected in 2013. While it is clear that within-region diversity was lowest in the North region in the most recent sampling year, that pattern was inconsistent in previous years. Among all regions except for the North region, there was clear support for genetic diversity within regions increasing over time

Within-region genetic diversity varied across space and time. Such a pattern may suggest that the relative age of tick populations varies among regions. For example, the pairwise genetic distances between mitochondrial sequences of ticks within the Hudson River Valley were generally higher than those of other regions, with the clearest support for this among ticks collected in 2013, suggesting tick populations from the Hudson River Valley may be the oldest in New York (Fig. 4). The greater diversity within the Hudson River Valley could also result from higher rates of migration into this region, among several other ecological or evolutionary factors. In contrast, the pairwise genetic distances between ticks in the North region were the lowest. While within-region diversity was lowest in the North region in the most recent sampling year (2017), that pattern was inconsistent in previous years. Among all regions except for the North region, diversity within regions appeared to be increasing over time.

### Population demography

**Fig. 5:**
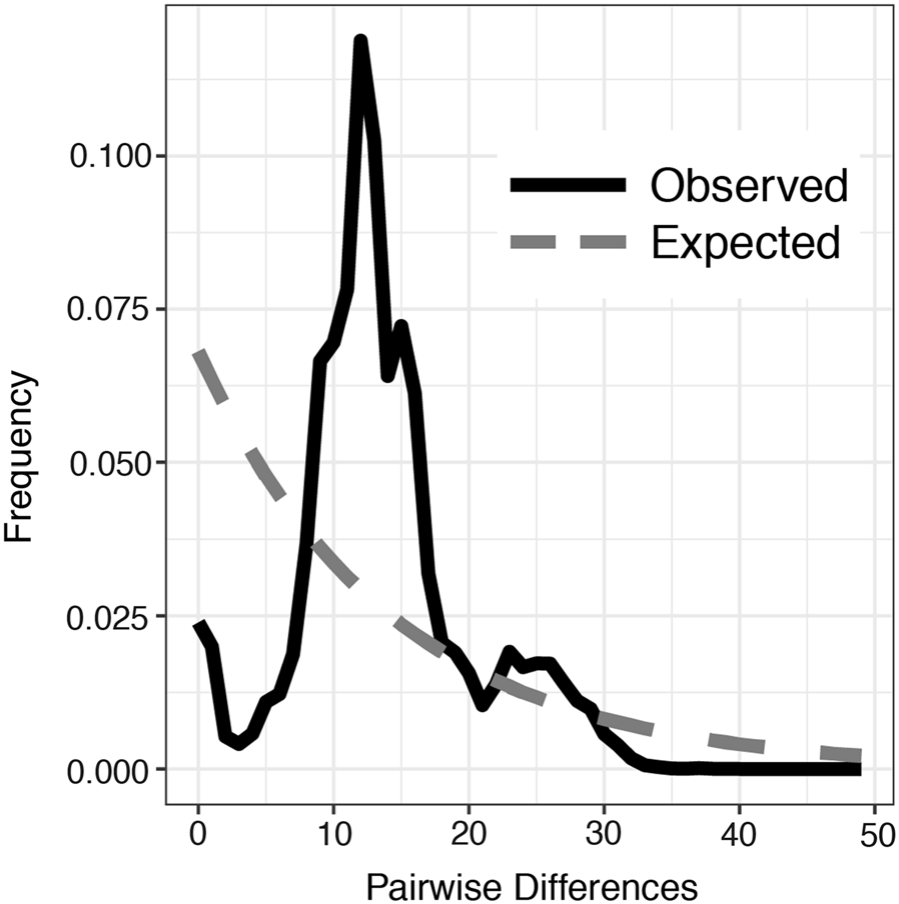
The observed frequency distribution of pairwise differences among haplotypes (solid line) of *Ixodes scapularis*differs from that expected in populations that are not expanding (dashed line). Unimodal frequency distributions are most commonly interpreted as indications of increasing population sizes

The blacklegged tick population size has increased dramatically in the last two decades. The distribution of mismatches found among all sampled ticks (Fig. 5) and among ticks within each region differed from the distribution that would be expected in populations experiencing no demographic changes. The unimodal distribution of pairwise differences is commonly interpreted as an increasing population size although they can also indicate increasing geographic range [62].

## Discussion

We detailed the population dynamics of the blacklegged tick (*Ixodes scapularis*) in New York during a time frame in which tick-borne diseases were increasing in range and incidence. We found evidence of population genetic structure at a broad geographic scale due to differences in the relative abundance, but not the composition, of haplotypes among sampled ticks (Figs. 2a, 3b; Table 2). Said otherwise, genetically similar ticks were found across New York, suggesting that populations across the state are connected by migration and colonization events, but these events do not occur with sufficient regularity to homogenize the genetic diversity among populations. Limited gene flow among regions could be caused by rare migration events between regions or rare colonization events due to ecological differences among regions. Limited gene flow resulted in a pattern of isolation by distance which was statistically supported when ticks from regions outside New York were included in analyses (Fig. 2b). Within most regions, the population genetic diversity increased over time, suggesting gene flow in the recent past. These data provide a foundation on which to generate testable hypotheses on the drivers of tick population dynamics occurring at finer scales.

Short- and long-range migration events are relatively common among ticks in New York. The active dispersal capabilities of ticks are minimal such that migration occurs primarily while attached to a vertebrate host [63–65]. Prior analyses of data from the Hudson River Valley (Hudson region) indicated that the probability that a tick population would colonize a novel location was correlated with how geographically close that location was to an already established population, consistent with gene flow via terrestrial vertebrate hosts [10]. The data presented here provide evidence of geographic signatures in phylogenetic relationships that support this conclusion; that is, ticks were often most closely related to ticks from the same and nearby collection sites. For example, ticks from the East Central region clustered together on the most probable phylogeny, and at a finer scale, ticks collected from Site 6 in the Hudson region are closely related to each other (Fig. 3c). Additionally, a Mantel test supports a correlation between genetic distance between ticks and geographic distance between the sites where those ticks were collected.

Longer-range gene flow has also occurred, as some ticks were most closely related to ticks from distant sites, although this appears to be less common than more proximal gene flow (Fig. 3c). These findings are consistent with those of other studies in the continental USA which have demonstrated that migrating birds impact tick range expansion and the maintenance of connectivity among tick populations at broad spatial scales [66, 67]. While an alternative cause of closely-related ticks from geographically distant areas could be multiple short-distance gene flow events by ticks attached to terrestrial reservoir hosts, the time frame represented by these data is too short to support this premise.

Analyses of this coarse scale dataset do not indicate the ecological or environmental processes that have driven tick range expansion across New York. Investigations of ticks migrating into Canada suggest that songbirds carry ticks northward beyond areas with climates suitable for tick populations [68, 69]. In such areas, tick populations cannot persist, and those areas are repeatedly colonized by migrating birds carrying ticks [68–70]. While birds are likely involved in dispersing ticks among locations within New York, there is no evidence that migration of tick-infested birds is driving tick range expansions. Differences in the drivers of ranges expansion within New York, which is not at the current northern boundary of the species range, and into Canada, which is at the species range boundary, should be expected as the processes impacting tick population dynamics are likely to vary among regions with different underlying ecologies and environmental conditions [71].

Survey-based surveillance efforts have revealed which landscape and climatic features drive tick presence and absence, tick densities and subsequent tick-borne disease risk [22, 72]. Mitochondrial genome sequencing allows for the study of gene flow between established populations, which is not possible with epidemiological survey data, to determine how environmental features impact current migration as well as range expansions. In the present study, mitochondrial genome sequencing revealed the role of short- and long-range migration events in the evolutionary history of ticks in New York. Analyses of entire mitochondrial genomes increase analytical power over many prior studies that use a few loci with limited genetic variation [10, 73], thus permitting investigations at fine spatiotemporal scales, the scale at which many ecological processes occur. For example, hypotheses that focus on identifying and quantifying the impact of environmental features on migration rates, colonization rates, population persistence and on other tick population dynamic characteristics can be tested.

## Conclusions

Understanding tick population dynamics is critical to the design of effective strategies to mitigate risk from tick-borne pathogens. Tick-borne pathogens require the establishment of populations of highly competent vectors, like *I. scapularis,* to occur at epidemiologically relevant levels in a given area [69, 74]. While there is evidence of some limitations to gene flow, tick migration occurs at both coarse and fine scales, which could have implications for further range expansion as habitat suitability for ticks changes due to global climate change [75, 76]. Analyses of mitochondrial genome sequencing data will expound upon previously identified drivers of tick presence and abundance and can further identify additional drivers. These analyses are critical to ascertain the connectivity among tick populations, how and why current gene flow occurs between established populations, and how environmental features likely impact these population dynamics.

## Supplementary Information

**Additional file 1.**: **Table S1. **Sample metadata. For each tick, this includes year collected, sex, and collection site information.

**Additional file 2.**: **Table S2. **Long-range PCR primer sequences.

**Additional file 3. **: **Figure S1. **Clusters of haplotypes (determined by K-means clustering) were represented across all regions for all values of K tested. Each row represent a different value for K (from K=6 to K=9). For each value of K, the proportion of each cluster (colored along a gray gradient) within each region is shown. 

## Data Availability

Sequence data that support the findings of this study were deposited in GenBank (Accession PRJNA825593).
