# Supplementary figures and images for: Phylogeographic dynamics of the arthropod vector, the blacklegged tick (Ixodes scapularis)
Source: Parasit Vectors. 2022 Jun 28;15:238. doi: 10.1186/s13071-022-05304-9 (PMC9241328; doi:10.1186/s13071-022-05304-9)

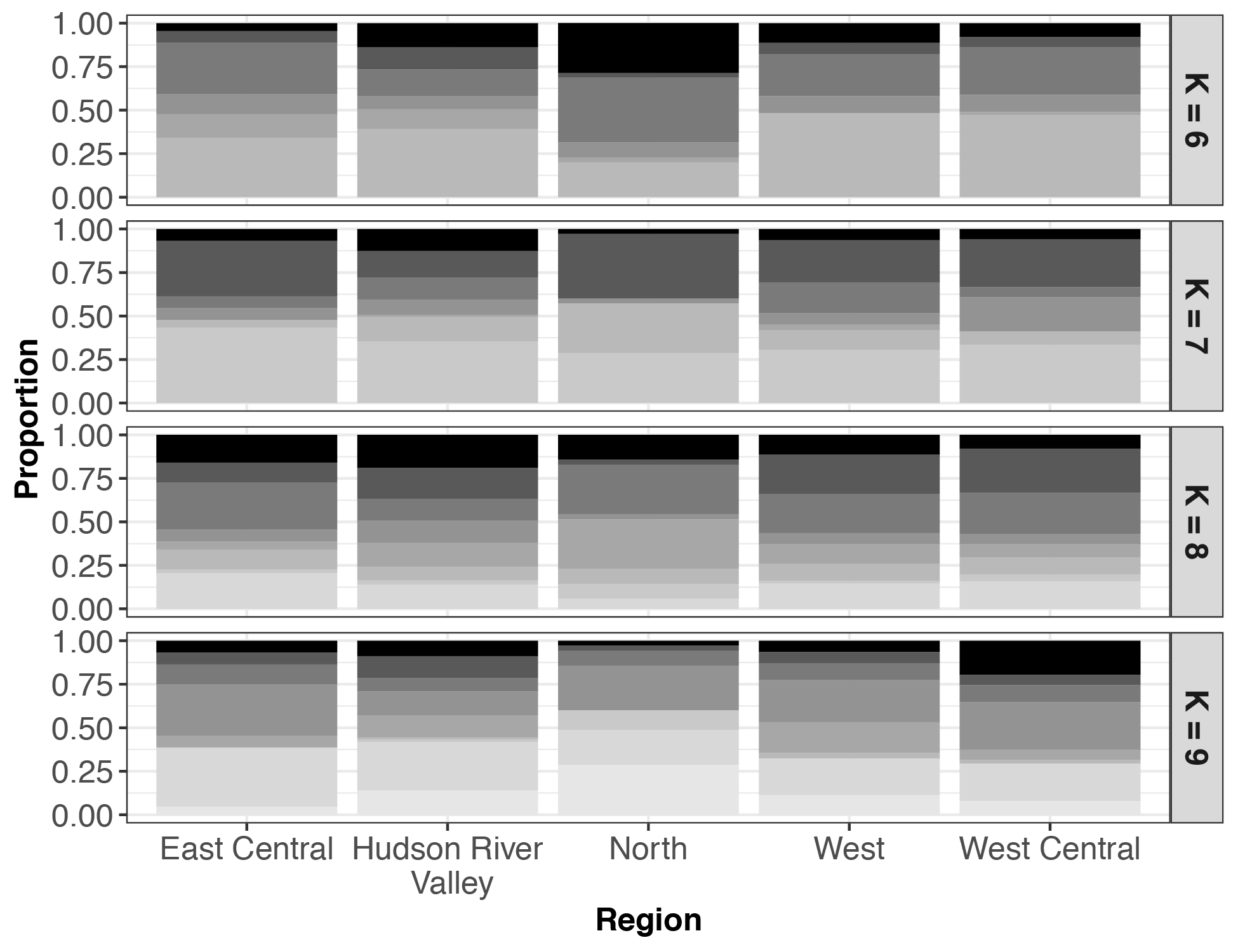

Supplement: Supplementary file 3 — Additional file 3. : Figure S1. Clusters of haplotypes (determined by K-means clustering) were represented across all regions for all values of K tested. Each row represent a different value for K (from K=6 to K=9). For each value of K, the proportion of each cluster (colored along a gray gradient) within each region is shown. [file 13071_2022_5304_MOESM3_ESM.png]
